# Neutrophil gelatinase–associated lipocalin as a biomarker of nephropathy in sickle cell disease

**DOI:** 10.1007/s00277-021-04500-4

**Published:** 2021-04-02

**Authors:** Rajaa Marouf, Adekunle D. Adekile, Hadeel El-Muzaini, Rasha Abdulla, Olusegun A. Mojiminiyi

**Affiliations:** 1 Department of Clinical Pathology, Faculty of Medicine, Kuwait University, PO Box 24923, Safat 13110, Kuwait City, Kuwait; 2 Department of Pediatrics, Faculty of Medicine, Kuwait University, PO Box 24923, Safat 13110, Kuwait City, Kuwait

**Keywords:** Sickle cell disease, Sickle cell nephropathy, Neutrophil gelatinase–associated lipocalin, Glomerular filtration rate, Urine microalbumin:creatinine ratio

## Abstract

Sickle cell nephropathy (SCN) develops via altered hemodynamics and acute kidney injury, but conventional screening tests remain normal until advanced stages. Early diagnostic biomarkers are needed so that preventive measures can be taken. This study evaluates the role of neutrophil gelatinase–associated lipocalin (NGAL) as a biomarker of SCN in steady state and vaso-occlusive crisis (VOC). In this case-control study, 74 sickle cell disease (SCD) patients (37 in steady state and 37 in VOC) and 53 control subjects had hematological and biochemical measurements including plasma and urine NGAL. Univariate and logistic regression analyses were used to find the associations between variables. The receiver operating characteristic (ROC) curve was used to determine the diagnostic performance characteristics of plasma and urine NGAL for detection of VOC. Plasma and urine NGAL, urine microalbumin:creatinine ratio, and urine protein:creatinine ratio were significantly higher in VOC. Microalbuminuria was present in 17.1% steady state and 32.0% VOC patients. Microalbuminuria showed significant correlations with age, plasma NGAL, WBC, and hemolytic parameters. Area under the ROC curve for plasma NGAL was 0.69 (95%CI = 0.567–0.813; *p* = 0.006) and 0.86 (95%CI = 0.756–0.954; *p* < 0.001) for urine NGAL. Urine NGAL cut-off value of 12.0 ng/mL had 95% sensitivity and 65% specificity. These results confirm the presence of nephropathy during VOC and suggest that plasma and urine NGAL would be useful in the identification of SCN. Urine NGAL should be used as the screening biomarker, and patients with VOC and urine NGAL > 12.0 ng/mL should be selected for aggressive management to prevent progression of renal damage.

## Introduction

Sickle cell disease (SCD) is an inherited hemoglobinopathy that is relatively prevalent in Kuwait and the neighboring countries. It is considered to be mild among Kuwaiti patients because of their β^S^ haplotype, high fetal hemoglobin (Hb F) level, and high prevalence of α-thalassemia [1].

Sickle cell nephropathy (SCN) is one of the recognized complications of SCD causing significant morbidity and mortality in patients with end-stage renal disease (ESRD). It affects nearly 30–50% of adults with SCD [2]. Median survival among patients with and without kidney failure was found to be 29 and 51 years, respectively [3]. Evidence-based guidelines for screening, diagnosis, and management of SCN are lacking.

Chronic hemolysis and vasculopathy are two proposed mechanisms for many complications of SCD including SCN [4]. The molecular mechanisms behind renal damage in SCD have not been fully identified, although the major underlying pathophysiology results from hypoxia, ischemia, and hemolysis [5]. SCN is characterized by hyperfiltration at a young age, which results in hyposthenuria. At this age, patients with SCD have supra-normal glomerular filtration rate (GFR), proximal tubular dysfunction, and impaired ability to acidify urine or excrete potassium [6]. Glomerulonephropathy tends to occur as patients grow older, leading to microalbuminuria. This progresses to macroalbuminuria (gross proteinuria) and eventually ESRD. Studies show that gross proteinuria and ESRD are observed in 15–30% of patients with SCD [2]. Renal tubular defects and hematuria are other manifestations of SCN. Conventional renal function tests like serum creatinine and GFR become abnormal only when renal damage has become extensive and largely irreversible.

Patients with SCD have increased GFR and high renal blood flow and, therefore, have high urine creatinine clearance. This makes estimations of GFR from creatinine clearance or serum creatinine inaccurate. A more reliable method of estimating GFR in patients with SCD is serum cystatin C that is freely filtered in glomeruli [7]. Micro and macroalbuminuria are other methods of detecting glomerulopathy in SCD.

There is a growing need to develop non-invasive and more sensitive markers for the early detection of renal dysfunction since derangement of the standard tests would be late indicators of irreversible kidney damage. Additionally, serum creatinine level is limited as a marker because of its variation as a function of muscle mass, gender, race, medications, and comorbid conditions. In patients with SCD, serum creatinine level is low due to increased excretion in the urine, and therefore, it rises only in the late stages of SCN.

Not all patients develop SCN. It is not clear how the recognized genetic factors that modulate SCD phenotype influence the progression of SCN in susceptible patients. Early detection of SCN is warranted so that prophylactic and therapeutic interventions can be introduced before renal damage occurs or becomes irreversible.

### Neutrophil gelatinase–associated lipocalin

Neutrophil gelatinase–associated lipocalin (NGAL), also known as lipocalin-2, is a recently identified adipokine that belongs to the superfamily of lipocalins. It is a glycoprotein involved in transmembrane transportation of lipophilic substances [8]. It is found in activated neutrophils and several other tissues including the liver, kidney, adipocytes, and macrophages. Kidney tubular cells may produce NGAL in response to various injuries. It is a newly recognized marker of nephropathy. NGAL was suggested to be a biomarker of acute kidney injury (AKI) even in the setting of chronic kidney disease. It has a strong association with albuminuria [9]. Although NGAL is normally present in the circulation, only a very small amount is expressed in the kidneys and excreted in the urine. Systemic NGAL is freely filtered through the glomerulus, but it is totally reabsorbed by the renal proximal tubules. Following ischemia and AKI, NGAL is one of the earliest substances that are released into the urine. Due to increased GFR in SCD, it is unclear whether the levels of this biomarker would be elevated or subnormal. Devarajan and coworkers have identified NGAL as a novel sensitive marker of renal tubular damage in acute and chronic nephropathy [10]. However, there are limited studies of NGAL in SCD as a marker of renal tubular damage and SCN [11].

The objective of this study was to evaluate the diagnostic performance characteristics of plasma and urine biomarkers of nephropathy in steady state and vaso-occlusive crisis (VOC) in SCD. Additionally, this study aimed to examine the association between SCN and markers of hemolysis, as well as the effect of Hb F and α-thalassemia on the presence and degree of nephropathy.

## Materials and methods

A case-control study of adult Kuwaiti patients with HbSS or HbS/ß^0^-thalassemia attending a hematology clinic at Mubarak Al-Kabeeer Hospital was conducted. They were in steady state, i.e., did not have any acute illness, VOC, or blood transfusion 3 weeks prior to enrollment. Another cohort of patients with acute VOC, defined as an episode of acute pain [12], were included within 24 h of admission. SCD was confirmed by Hb electrophoresis.

Patients were excluded if they had diabetes mellitus, malignancy, or receiving angiotensin-converting enzyme inhibitors or angiotensin II receptor blocker or on a blood transfusion program.

Age- and ethnicity-matched healthy controls were recruited from blood donors and their relatives. All participants gave written informed consent prior to their inclusion in the study in accordance with the ethical standards of the Helsinki Declaration [13]. Ethical approval of the study protocol was also granted by the Health Sciences Center Ethical Committee, Kuwait University.

Frequency of VOC, blood transfusion history, and complications of SCD data were collected from patients. Age, gender, height, weight, blood pressure data and blood and early morning urine were collected from patients and controls.

Hemoglobin, WBC, platelets, reticulocyte count, and Hb F were determined on UniCel DxH 800, Coulter Cellular Analysis System (Beckman-Coulter, CA, USA) and Bio-Rad VARIANT II Instrumentation (CA, USA).

Renal function tests (electrolytes, urea, and creatinine), liver function tests (bilirubin, ALT, ALP, GGT), lactate dehydrogenase, and haptoglobin were determined on Beckman DXC 800 (Beckman-Coulter, CA, USA).

Estimated GFR (eGFR) was calculated using the Chronic Kidney Disease Epidemiology Collaboration creatinine equation (2009) (eGFR_creat_) [14].

Cystatin C was measured on Cobas 501C analyzer (Roche Diagnostics, Rotkreuz, Switzerland). eGFR was calculated using the Chronic Kidney Disease Epidemiology Collaboration cystatin C equation (2012) (eGFR_cys_) [15].

Urine microalbumin concentration was measured on an Immage 800 analyzer (Beckman Corporation, CA, USA). Urine creatinine and protein were determined with the Beckman Synchron DXC 800 automated analyzer (Beckman Corporation, CA, USA).

Normoalbuminuria and microalbuminuria were defined as urine microalbumin:creatinine ratio < 30 mg/g and 30–300 mg/g respectively [16].

Plasma and urine NGAL were measured using the NGAL Test Reagent Kit (BioPorto Diagnostics A/S, Denmark) on a Cobas 501C analyzer (Roche Diagnostics, Rotkreuz, Switzerland) after evaluation of assay.

Alpha-thalassemia testing was done by polymerase chain reaction using an ABI 9700 thermal cycler (Thermo Fisher Scientific, MA, USA) utilizing forward and reverse primers to detect the − α^3.7kb^ deletion [17], which is the most common α-thalassemia allele in the region [18].

### Statistical analysis

Statistical analysis was performed using IBM SPSS, version 25 (Chicago, IL, USA). Non-parametric quantitative data were presented as median (interquartile range), while categorical data were presented as frequencies (%). Comparison of categorical data was made using Pearson’s chi-square test, while for quantitative data, Mann-Whitney *U* and Kruskal-Wallis tests were used. Spearman’s rank correlation coefficient was used to investigate the correlation between various parameters. Odds ratio (OR) and 95% confidence interval (95% CI) were determined by binary logistic regression. Receiver operating characteristic (ROC) curve analysis was used to determine the diagnostic performance characteristics of the test. The level of significance was set at *p* < 0.05.

## Results

A total of 80 patients with SCD (HbSS and HbS/ß-thalassemia) were recruited. Six patients were excluded due to comorbidities stated above. Seventy-four patients were included in the analysis. Thirty-seven patients (14 males and 23 females) were in steady state, and 37 (18 males and 19 females) were in VOC. Fifty-three (12 males and 41 females) control subjects were analyzed.

The demographic and clinical data for the study subjects are summarized in Table 1. Age, hydroxyurea treatment, and alpha-globin genotype frequencies were not significantly different between patients in steady state and those in VOC. Hematological and biochemical parameters showed the expected variabilities between patients in steady state and those in VOC with WBC, reticulocyte count, lactate dehydrogenase, ferritin, and erythropoietin being higher in patients in VOC.

**Table 1:** Demographic, clinical, and laboratory data of the study population. Results are presented as median (interquartile range) unless stated otherwise

	Controls (*n* = 53)		Steady state patients (*n* = 37)		VOC patients (*n* = 37)		*p* value (comparing all groups)	*p* value (comparing steady state and VOC)
Gender (% female)	41	(77.4%)	23	(62.2%)	19	(51.4%)	**0.034**^**a**^	0.348^a^
Frequency of VOC in past 5 years (*n* (%))								0.138^a^
<1 episode/year			17	(47.2%)	9	(28.1%)		
1–3 episodes/year			10	(27.8%)	8	(25.0%)		
> 3 episodes/year			9	(25.0%)	15	(46.9%)		
History of blood transfusions (*n* (%))								0.002^a^
0–3			29	(80.6%)	17	(45.9%)		
> 3			7	(19.4%)	20	(54.1%)		
Alpha-globin genotype (% yes)			21	(63.6%)	21	(75.0%)		0.340^a^
Hydroxyurea Rx (% yes)			13	(35.1%)	13	(36.1%)		0.931^a^
Age (years)	28.0	(18.0)	31.0	(24.0)	30.0	(11.5)	0.706^b^	
BMI	26.9	(6.4)	25.1	(7.8)	25.5	(6.1)	0.409^b^	
Blood pressure—systolic (mmHg)	115.0	(16.5)	125.0	(19.3)	124.5	(14.5)	**0.015**^**b**^	0.421^c^
Blood pressure—diastolic (mmHg)	73.0	(12.0)	77.0	(14.5)	67.5	(14.3)	**0.002**^**b**^	**0.002**^**c**^
Heart rate (bpm)	79.0	(16.0)	84.0	(17.0)	92.0	(16.0)	**0.009**^**b**^	0.117^c^
White blood cell count (× 10^9^/L)	6.6	(2.7)	7.3	(5.4)	12.7	(10.0)	**0.000**^**b**^	**0.000**^**c**^
Hemoglobin (g/L)	129.0	(14.0)	112.0	(17.0)	95.0	(34.0)	**0.000**^**b**^	**0.002**^**c**^
Platelet count (× 10^9^/L)	280.0	(77.5)	236.0	(220.5)	316.0	(297.0)	0.604^b^	0.570^c^
Reticulocyte absolute count (× 10^12^/L)	0.0550	(0.0230)	0.1745	(0.0970)	0.2140	(0.1005)	**0.000**^**b**^	0.069^c^
Total bilirubin (μmol/L)	12.0	(6.2)	35.7	(24.1)	34.9	(18.7)	**0.000**^**b**^	0.655 ^c^
Direct bilirubin (μmol/L)	2.0	(1.0)	5.0	(2.0)	6.0	(4.0)	**0.000**^**b**^	0.092^c^
Lactate dehydrogenase (IU/L)	128.0	(36.0)	240.0	(89.0)	325.0	(253.5)	**0.000**^**b**^	**0.000**^**c**^
Ferritin (ng/mL)	22.8	(46.2)	106.3	(221.1)	393.9	(494.6)	**0.000**^**b**^	**0.000**^**c**^
Haptoglobin (g/L)	1.26	(0.54)	0.06	(0.00)	0.06	(0.00)	**0.000**^**b**^	0.655^c^
Erythropoietin (mIU/L)	9.0	(4.9)	42.7	(52.6)	67.3	(133.6)	**0.000**^**b**^	**0.045**^**c**^
Serum urea (mmol/L)	3.6	(1.4)	2.6	(0.7)	3.1	(1.7)	**0.003**^**b**^	0.222^c^
Serum creatinine (μmol/L)	56.0	(19.0)	54.0	(17.5)	58.4	(26.7)	**0.049**^**b**^	0.132^c^
Cystatin C (mg/L)	0.87	(0.15)	0.93	(0.18)	0.94	(0.45)	0.106^b^	0.809^c^
eGFR_creat_ (mL/min/1.73 m^2^)	119.6	(23.3)	127.6	(27.6)	129.8	(40.2)	**0.048**^**b**^	0.570^c^
eGFR_cys_ (mL/min/1.73 m^2^)	99.0	(21.0)	93.5	(31.1)	98.3	(59.4)	0.304^b^	0.803^c^
Urine microalbumin:creatinine ratio (mg/g)	5.0	(3.0)	6.0	(12.0)	16.0	(73.0)	**0.000**^**b**^	**0.011**^**c**^
Urine protein:creatinine ratio (g/g)	0.045	(0.023)	0.067	(0.048)	0.181	(0.386)	**0.000**^**b**^	**0.000**^**c**^
Plasma NGAL (ng/mL)	83.0	(46.0)	97.0	(104.3)	129.0	(131.0)	**0.001**^**b**^	**0.006**^**c**^
Urine NGAL (ng/mL)	6.0	(13.0)	10.0	(14.3)	34.5	(62.3)	**0.000**^**b**^	**0.000**^**c**^

*VOC* vaso-occlusive crisis, *eGFR*_*creat*_ creatinine-based estimated glomerular filtration rate, *eGFR*_*cys*_ cystatin C-based estimated glomerular filtration rate, *NGAL* neutrophil gelatinase–associated lipocalin

^a^*p* value calculated by Pearson’s chi-square test

^b^*p* value calculated by Kruskal-Wallis test

^c^*p* value calculated by Mann-Whitney *U* test

Significant *p* values indicated in bold

### Markers of renal involvement during VOC

Plasma and urine NGAL were significantly increased in patients in VOC compared to those in steady state and controls (Table 1). Urine microalbumin:creatinine ratio and urine protein:creatinine ratio showed a similar trend with the highest levels found in patients with VOC. Although the Kruskal-Wallis analysis of variance showed significant trends in markers of glomerular filtration (serum urea, serum creatinine, eGFR_creat_), the Mann-Whitney *U* test showed that these markers could not be used to differentiate patients in steady state from those in VOC (serum urea (*p* = 0.222), serum creatinine (*p* = 0.132), and eGFR_creat_ (*p* = 0.570)). Serum cystatin C (*p* = 0.106) and eGFR_cys_ (*p *= 0.304) did not show significance in distinguishing controls from patients in steady state and those in VOC. These observations may be due to the fact that the patients studied had relatively normal renal function.

Further clinical and laboratory parameters of the patients grouped by presence or absence of microalbuminuria are shown in Table 2. None of the control subjects had microalbuminuria, whereas 17.1% of subjects in steady state and 32.0% of subjects in VOC had microalbuminuria (*p* = 0.180). Age, WBC, reticulocyte count, total bilirubin, lactate dehydrogenase, ferritin, and erythropoietin were significantly higher in patients with microalbuminuria, whereas Hb and haptoglobin were significantly lower. Binary logistic regression analysis showed that the significant factors associated with microalbuminuria in these patients were age (OR = 1.06; 95% CI = 1.02–1.11, *p* = 0.007), plasma NGAL (OR = 1.01; 95% CI = 1.00–1.02; *p* = 0.009), and urine protein:creatinine ratio (OR = 344.27; 95% CI = 2.77–42,848.33; *p* = 0.018). Surprisingly, urine NGAL did not prove to be a significant determinant of microalbuminuria (OR = 1.01; 95% CI = 1.00–1.03; *p* = 0.143) as were markers of GFR (data not shown).

**Table 2:** Clinical and laboratory parameters of the patients grouped by presence or absence of microalbuminuria. Results are presented as median (interquartile range)

Urine microalbumin:creatinine ratio (mg/g)	< 30		> 30		*p* value^a^
Age (years)	30.0	(15.0)	43.0	(33.5)	**0.026**
Blood pressure—systolic (mmHg)	118.0	(18.0)	133.0	(17.0)	**0.000**
Blood pressure—diastolic (mmHg)	73.0	(12.0)	80.0	(20.0)	0.167
White blood cell count (× 10^9^/L)	7.0	(3.9)	11.3	(8.2)	**0.010**
Hemoglobin (g/L)	125.0	(22.0)	107.5	(35.8)	**0.003**
Platelet count (× 10^9^/L)	274.0	(154.0)	247.0	(191.5)	0.568
Reticulocyte absolute count (× 10^12^/L)	0.0780	(0.1150)	0.2060	(0.1140)	**0.000**
Total bilirubin (μmol/L)	18.0	(21.8)	34.1	(18.6)	**0.001**
Direct bilirubin (μmol/L)	3.0	(3.1)	5.0	(4.5)	**0.001**
Lactate dehydrogenase (IU/L)	160.0	(115.0)	304.5	(147.3)	**0.000**
Ferritin (ng/mL)	54.7	(108.5)	409.3	(2562.0)	**0.000**
Haptoglobin (g/L)	0.9	(1.2)	0.1	(0.0)	**0.005**
Hb F (%)	18.05	(7.8)	15.2	(14.3)	0.274
Erythropoietin (mIU/L)	14.8	(40.8)	49.2	(77.5)	**0.002**
Serum urea (mmol/L)	3.1	(1.4)	3.3	(2.7)	0.301
Serum creatinine (μmol/L)	56.0	(16.0)	61.5	(25.5)	0.268
Cystatin C (mg/L)	0.89	(0.16)	0.96	(0.44)	0.197
eGFR_creat_ (mL/min/1.73 m^2^)	123.8	(25.2)	102.6	(63.9)	0.241
eGFR_cys_ (mL/min/1.73 m^2^)	99.0	(22.6)	95.1	(57.6)	0.392
Urine protein:creatinine ratio (g/g)	0.049	(0.038)	0.344	(1.561)	**0.000**
Plasma NGAL (ng/mL)	89.0	(69.0)	136.0	(186.5)	0.078
Urine NGAL (ng/mL)	9.0	(17.5)	19.0	(36.0)	**0.009**

*Hb F* fetal hemoglobin, *eGFR*_*creat*_ creatinine-based estimated glomerular filtration rate, *eGFR*_*cys*_ cystatin C-based estimated glomerular filtration rate, *NGAL* neutrophil gelatinase–associated lipocalin

^a^*p* value calculated by Mann-Whitney *U* test

Significant *p* values indicated in bold

**Fig. 1:**
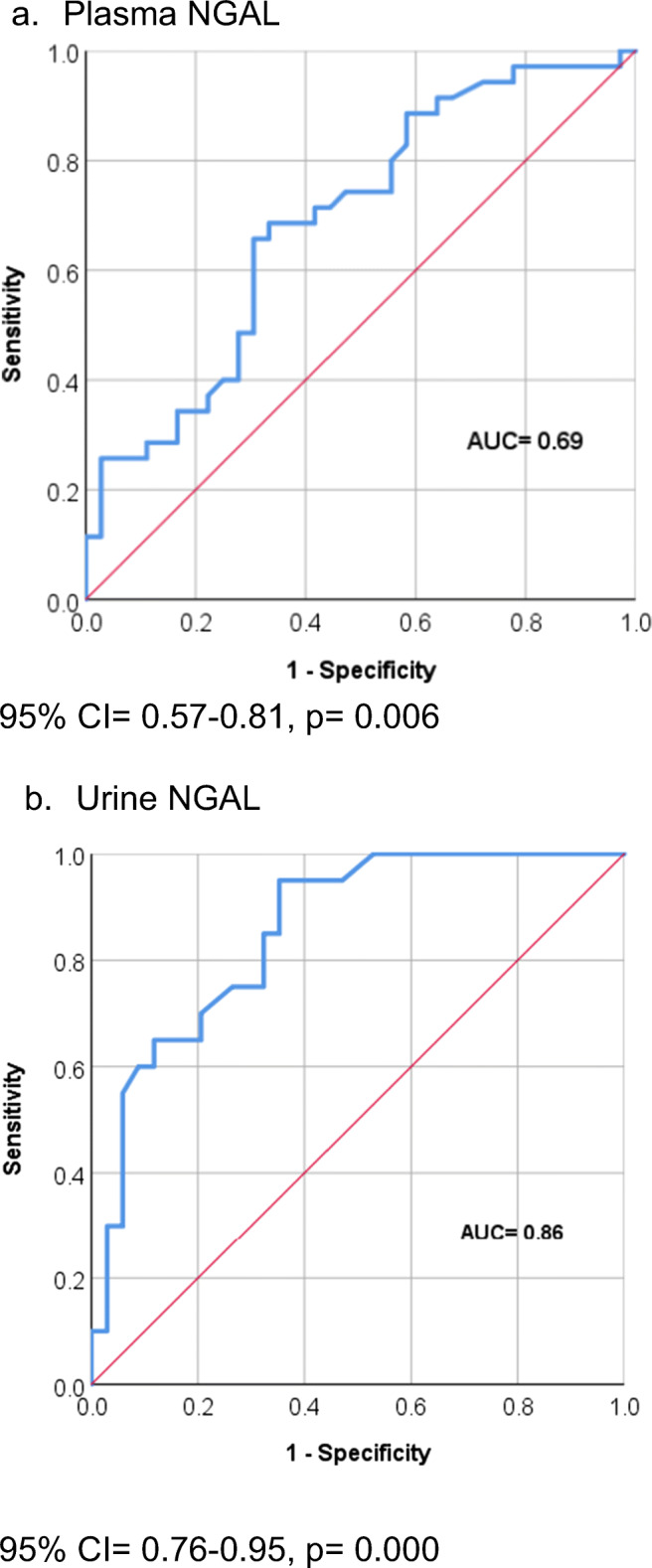
Receiver operating characteristic (ROC) analyses comparing the diagnostic potential of plasma and urine NGAL in identifying patients with vaso-occlusive crisis. NGAL neutrophil gelatinase–associated lipocalin, AUC area under the curve

Age showed significant (*p* < 0.05) correlations with serum urea (*r* = 0.39), serum creatinine (*r* = 0.29), eGFR_creat_ (*r* = − 0.75), serum cystatin C (*r* = 0.19), and eGFR_cys_ (*r* = − 0.36). Age did not significantly correlate with plasma and urine NGAL, urine microalbumin:creatinine ratio, and urine protein:creatinine ratio. Plasma and urine NGAL showed variable correlations with hematological and biochemical parameters (Table 3). Binary logistic regression suggests that urine NGAL (OR = 1.05; 95% CI = 1.02–1.09, *p* = 0.004) may be the slightly better marker than plasma NGAL (OR = 1.01; 95% CI = 1.00–1.02; *p* = 0.011). ROC analyses (shown in Fig. 1a, b) were performed to compare the diagnostic potentials of plasma and urine NGAL in identifying patients with VOC. Both markers showed fairly good diagnostic potentials with area under the curve (AUC) for plasma NGAL of 0.69 (95% CI = 0.567–0.813; *p* = 0.006) and of 0.86 for urine NGAL (95% CI = 0.756–0.954; *p* < 0.001). The best cut-off value of urine NGAL was 12.0 ng/mL, with a sensitivity of 95% and specificity of 65%.

**Table 3:** Correlation coefficients of plasma NGAL and urine NGAL with hematological and biochemical parameters

	Plasma NGAL (ng/mL)	Urine NGAL (ng/mL)
Age (years)	0.145	0.047
BMI	0.207*	0.053
White blood cell count (× 10^9^/L)	0.439**	0.108
Hemoglobin (g/L)	− 0.227*	− 0.373**
Platelet count (× 10^9^/L)	0.108	− 0.165
Reticulocyte absolute count (× 10^12^/L)	0.310**	0.184
Lactate dehydrogenase (IU/L)	0.313**	0.303**
Haptoglobin (g/L)	− 0.137	− 0.226*
Ferritin (ng/mL)	0.125	0.384**
Hb F (%)	− 0.110	0.067
Serum urea (mmol/L)	− 0.068	− 0.038
Serum creatinine (μmol/L)	0.131	0.035
Cystatin C (mg/L)	0.288**	0.043
eGFR_creat_ (mL/min/1.73 m^2^)	− 0.162	− 0.068
eGFR_cys_ (mL/min/1.73 m^2^)	− 0.310**	− 0.023
Urine microalbumin:creatinine ratio (mg/g)	0.284**	0.272**
Urine protein:creatinine ratio (g/g)	0.215*	0.414**
Urine NGAL (ng/mL)	0.243*	1.000

*Hb F* fetal hemoglobin, *eGFR*_*creat*_ creatinine-based estimated glomerular filtration rate, *eGFR*_*cys*_ cystatin C-based estimated glomerular filtration rate, *NGAL* neutrophil gelatinase–associated lipocalin

*Correlation is significant at the 0.05 level (2-tailed)

**Correlation is significant at the 0.01 level (2-tailed)

Significant *p* values indicated in bold

## Discussion

It is estimated that about one-third of patients with SCD develop insidious renal involvement that is similar in several aspects to the nephropathy that accompanies diabetes mellitus [19]. As occurs in diabetic nephropathy (DN), progressive changes occur in the kidneys resulting in hyperfiltration, development of microalbuminuria and macroalbuminuria, and progressive reduction of GFR and ESRD. However, unlike in DN where there are established guidelines on screening tests and therapeutic target guidelines, finding diagnostic and prognostic biomarkers and indications for therapeutic intervention in SCN remains challenging. In this comparative analysis of the performance characteristics of plasma and urine biomarkers of nephropathy, we have found that markers of GFR (serum creatinine, eGFR_creat_, cystatin C, eGFR_cys_) are not as useful as newer markers of AKI (plasma and urine NGAL) in the detection of renal involvement during VOC.

The significantly higher plasma and urine NGAL in patients with VOC compared to patients in steady state (Table 1) support the use of these biomarkers as indicators of renal involvement during VOC. However, different mechanisms are involved in the increased levels of these markers. Recurrent episodes of ischemia and ischemia-reperfusion injury during VOC are some of the main mechanisms that predispose to the development of recurrent AKI and SCN [20]. The significant correlation of WBC with plasma NGAL with the lack of correlation of urine NGAL suggests that the increase in plasma NGAL is due in part to the inflammatory processes that accompany VOC. A similar correlation was found between ferritin and urine NGAL (Table 3). After release by inflammatory processes, NGAL, a marker of neutrophil activation, is freely filtered in the glomeruli and reabsorbed in the proximal tubule by endocytosis. As our patients had normal renal glomerular filtration function, the increased plasma NGAL could also be due, in part, to tubular back leak of NGAL into the circulation due to the tubulo-interstitial damage that occurs during VOC [21]. Involvement of the renal tubules during VOC is supported by the significantly higher urine NGAL in patients with VOC compared to patients in steady state (Table 1) and the correlations of urine NGAL with hematological parameters (Table 3). Nevertheless, the correlations of both plasma and urine NGAL with hematological parameters in VOC suggest renal involvement in SCD patients with apparently normal renal function.

Clear-cut nephropathy was not found in any of our patients whether in steady state or VOC; however, the prevalence of microalbuminuria was 17.1% of subjects in steady state and 32.0% of subjects in VOC. This is much lower compared to other studies [21].

In the few other studies in our geographic region that addressed SCN, gross proteinuria (macroalbuminuria) was the parameter measured; however, more sensitive biomarkers such as microalbumin:creatinine ratio, plasma, and urine NGAL would better reflect renal dysfunction at an earlier stage [22, 23].

Our study shows that serum creatinine and urine microalbumin excretion, traditional markers of SCN, have variable usefulness in the detection of renal involvement during VOC. Our results on cystatin C were somewhat unexpected, as previous studies have shown that cystatin C is a superior marker of GFR in SCD [24]. Age was the main determinant of GFR in our cohort, and there was a poor correlation between hematological parameters and markers of GFR. The pathophysiologic changes in SCN are known to begin at an early age with progressive age-dependent worsening of renal function resulting from repetitive damage to glomerular and tubulo-interstitial compartments. Microalbuminuria, an early marker of glomerular dysfunction, is known to be a predictor of the development of ESRD. It is highly probable that, as happens in DN, renal tubular damage may precede the onset of microalbuminuria in the absence of significant glomerular damage that affects the GFR. The lack of significant differences in markers of GFR between patients with and without microalbuminuria (Table 2) supports this point. The finding of significantly higher urine NGAL with lack of significant difference in plasma NGAL indicates the differences in the mechanisms of the increases between the two biomarkers.

Mean Hb F level in the patient cohort was 18.6%. There was no correlation between Hb F and urine microalbumin:creatinine ratio, urine protein:creatinine ratio, plasma, and urine NGAL. Despite relatively high Hb F levels in our patients, it does not seem to be protective against SCN.

The presence of α-thalassemia trait was not different between steady state and VOC patients. Additionally, there was no association between the presence or absence of α-thalassemia trait and renal biomarkers.

Our results raise the question of the best biomarker that should be routinely used to detect renal involvement during VOC. ROC analysis (shown in Fig. 1) indicates that urine NGAL is better than plasma NGAL. There is abundant literature evidence that shows urine NGAL to be a diagnostic marker of acute tubular damage in various non-SCD diseases that cause AKI [25, 26]. Furthermore, urine NGAL has been shown to indicate the severity of renal involvement in various pathological states and predict the onset of acute renal failure [27]. On the basis of these findings, we suggest that urine NGAL should be measured routinely in patients with VOC and evaluated longitudinally to confirm its usefulness as an early biomarker of the progression of SCN. Early detection and appropriate treatment would reduce morbidity and mortality in patients with SCD.

In our study, we have shown that SCN is present in steady state and there is evidence of renal involvement during VOC. The study subjects had normal renal function as assessed by traditional markers. This group of patients would not have been viewed as having renal involvement especially during VOC. Additionally, we have shown that newer markers of acute kidney injury could indicate the presence of renal involvement especially during VOC. Using ROC analysis, we identified the best marker for screening for renal involvement and suggested an appropriate cut-off value. Our results show the need for screening for renal involvement during VOC even in subjects with apparently normal renal function. Although more clinical and translational research is needed to develop guidelines, we make the case for the need for appropriate guidelines that will aid in the screening, early diagnosis, and management of SCN, similar to what is available for diabetic nephropathy.

This study has some limitations. The study was not designed to evaluate AKI in patients with VOC as defined by the International Kidney Disease Improving Global Outcomes (KDIGO) guideline. Therefore, although serial serum creatinine estimations were made, patients were not stratified according to presence or absence or severity of AKI. Secondly, as our patients had relatively normal glomerular filtration function, the patients included probably have mild renal perturbations compared to studies in other centers that may have included patients with impaired renal function.

## Conclusion

VOC causes renal pathophysiological changes that may result in development of SCN if not detected, monitored, and treated in the long term. Urine NGAL should be used as the screening and diagnostic biomarker among other known biomarkers. Our results suggest that patients with VOC and urine NGAL > 12.0 ng/mL should be selected for more aggressive management to prevent progression to SCN. However, longitudinal studies are required to define evidence-based diagnostic and target therapeutic cut-off points.

In total, 17.1% of subjects in steady state and 32.0% of subjects in VOC had microalbuminuria. This low prevalence could not be explained by higher Hb F levels or presence of α- thalassemia trait. Other risk factors may contribute to this important finding.

## Data Availability

The datasets generated during and/or analyzed during the current study are available from the corresponding author on reasonable request.

## Code availability

Available from the corresponding author on reasonable request.
